# Influence of Large Reservoir Operation on Water-Levels and Flows in Reaches below Dam: Case Study of the Three Gorges Reservoir

**DOI:** 10.1038/s41598-017-15677-y

**Published:** 2017-11-15

**Authors:** Yunping Yang, Mingjin Zhang, Lingling Zhu, Wanli Liu, Jianqiao Han, Yanhua Yang

**Affiliations:** 1 Key Laboratory of Engineering Sediment, Tianjin Research Institute for Water Transport Engineering, Ministry of Transport, Tianjin, 300456 China; 2 State Key Laboratory of Water Resources and Hydropower Engineering Science, Wuhan University, Wuhan, 430072 China; 3 Bureau of Hydrology Changjiang Water Resources Commission, Wuhan, 430010 China; 4 Institute of Soil and Water Conservation, Northwest Agriculture and Forestry University, Yangling, 712100 China

## Abstract

The Three Gorges Project (TGP) is the world’s largest water conservation project. The post-construction low-flow water level at the same discharge below the dam has declined, but there remains disagreement over whether the flood level has increased. Measured water levels and upstream and downstream flow data from 1955 to 2016 show that, post-construction: (1) the low-flow water level at the same discharge decreased, and the lowest water level increased due to dry-season reservoir discharge; (2) the decline of the low-flow water level below the dam was less than the undercutting value of the flow channel of the river; (3) the flood level at the same discharge below the dam was slightly elevated, although peak water levels decreased; (4) flood characteristics changed from a high discharge–high flood level to a medium discharge – high flood level; and (5) an expected decline in the flood level downstream was not observed. Channel erosion and the adjustment of rivers and lakes tend to reduce flood levels, while river bed coarsening, vegetation, and human activities downstream increase the flood level. Although the flood control benefits of the Three Gorges Dam (TGD) and the upstream reservoirs are obvious, increased elevation of the downstream flood level remains a concern.

## Introduction

The huge storage capacity of a river-reach reservoir changes downstream flows and water quantities, intercepts downstream transportation of sediment, and leads to river erosion, water-level adjustments and other impacts, as stream reaches below the dam adapt to the discharge change and the sharp reduction in sediment supply. After construction of the Aswan Dam on the Nile River in Egypt, the average river channel undercut was 0.45 m, and the flow level at the same discharge showed a declining trend^1^. After the establishment of a reservoir on the Missouri River in the United States, the low water level dropped by more than 2.5 m, and the flood level increased by almost 1 m in the lower reaches around Kansas City^2^. In 2011, the Mississippi River flooded with a discharge lower than that measured during the 1927 and 1973 floods^3^, but due to the backwater caused by dense vegetation in higher parts of the floodplain, the flood level increased^4^, resulting in conditions of medium discharge but a high water level. Following r construction of the Danjiangkou Reservoir on the Hanjiang River in China, when the downstream discharge at Huangjiang port and Xiangfan Hydrological Station is less than 5,000 m^3^/s, the water level decreaseds by 1.5–1.7 m relative to pre-construction levels. When the discharge exceeds 10,000 m^3^/s, however, water levels are not significantly reduced compared to pre-construction values^5^. A review of the literature^6,7^ indicates that following the construction of a reservoir, the low-water level below the dam decreases at the same discharge, while the flood level at the same discharge does not change significantly or increases only slightly, although this will vary due to the different hydraulic factors for different rivers.

The Three Gorges Project (TGP) is the largest comprehensive water conservation project in the world^8^. It has considerable comprehensive benefits, such as flood control, power generation, shipping, water supply, and energy savings^9^. The influence of its construction and operation on the regulation of the downstream river, flood water levels and other important factors has attracted significant attention from researchers. The low-flow water level downstream of the Three Gorges Dam (TGD) is decreasing^10,11^, which is consistent with predictions^12,13^ and also with the decline of the low-flow water level downstream of the world’s other large reservoirs^6,7^. At these large reservoirs, downstream flood levels generally increase only slightly or do not change significantly^6,7^. As of yet, there is no common understanding of the variation in the downstream flood level before and after construction of the TGD, and it is not certain whether data show a decrease in the downstream flood level^14^,a small or non-significant increase^15^, or a rising trend^16,17^.

Recent studies have shown that, at times when the river downstream of the TGD was actively eroding, the flood level was not reduced. Instead, the flood diversion from the Jingjiang reach to Dongting Lake decreased, and the flood discharge capacity declined further^16^. Comparing the 2003–2013 and 2000–2002 periods, when the flow rate at Hankou Station was 50,000 m^3^/s, the corresponding increase in the water level was 0–1.0 m^17^. During July 2016, flood control water levels were exceeded at the lower sections of Jianli Station in the middle and lower reaches of the Yangtze River, and also at Dongting Lake and Poyang Lake. The total alarm period was 12 to 29 days, the longest since 1999, and in the reach from Hankou to Datong reach and Poyang Lake, the water level was the highest measured since 1999^18^. In 1954 and 1998, the maximum flow rate at Luoshan Station was 78,800 m^3^/s and 67,800 m^3^/s, respectively, and the corresponding water levels were 33.17 m and 34.95 m. Comparing 1954 and 1998, the maximum flow rate had decreased by 11,000 m^3^/s, and the water level had increased by 1.78 m^19^. In 2016, the maximum flow rate at Luoshan Station was 52,100 m^3^/s and the corresponding water level was 34.86 m, indicating that the flow rate had decreased by 15,700 m^3^/s and the water level had decreased by 0.09 m in comparison with those of 1998. After construction of the Three Gorges Reservoir (TGR), a consensus was reached that the lowest water level at the same discharge had decreased. Although it remains controversial whether the flood level has increased, it is necessary to study the characteristics, regularity, and genesis of the changes of the flood level downstream of the TGD. In general, siltation of the shoreline, decline of the branch river, shrinkage of the flood channel, and increased resistance in the river channel caused by coarsening of the river bed and encroachment of vegetation into the river channel are the main factors affecting flood-level elevation^20,21^. Following construction of the TGR, the erosion downstream of the dam intensified^22^, and the impact of factors, such as the coarsening of the river bed^23,24^, the variation in the number of days of floodplain inundation^23^, and the effects of human activities^25,26^, on the flood level is apparently increasing. Therefore, it is very important to conduct the relevant analysis as soon as possible.

The present study uses measured data from the period 1955–2016 to analyse the variation and regularity of the flood level and the low-flow water level below the TGD and to discuss the underlying causes, clarifying the relationship between changes in the low-flow water level and the increase of waterway depth, and the relationship between the change in the flood level and flood-control scenarios. The results obtained in this study provide important reference points for further optimization of the joint control of the TGR and the upstream cascade reservoirs, and prediction of the flood-control scenarios, well in advance of flood events.

## Study area and dispatching process of the runoff at the TGR

### Study area

**Figure 1:**
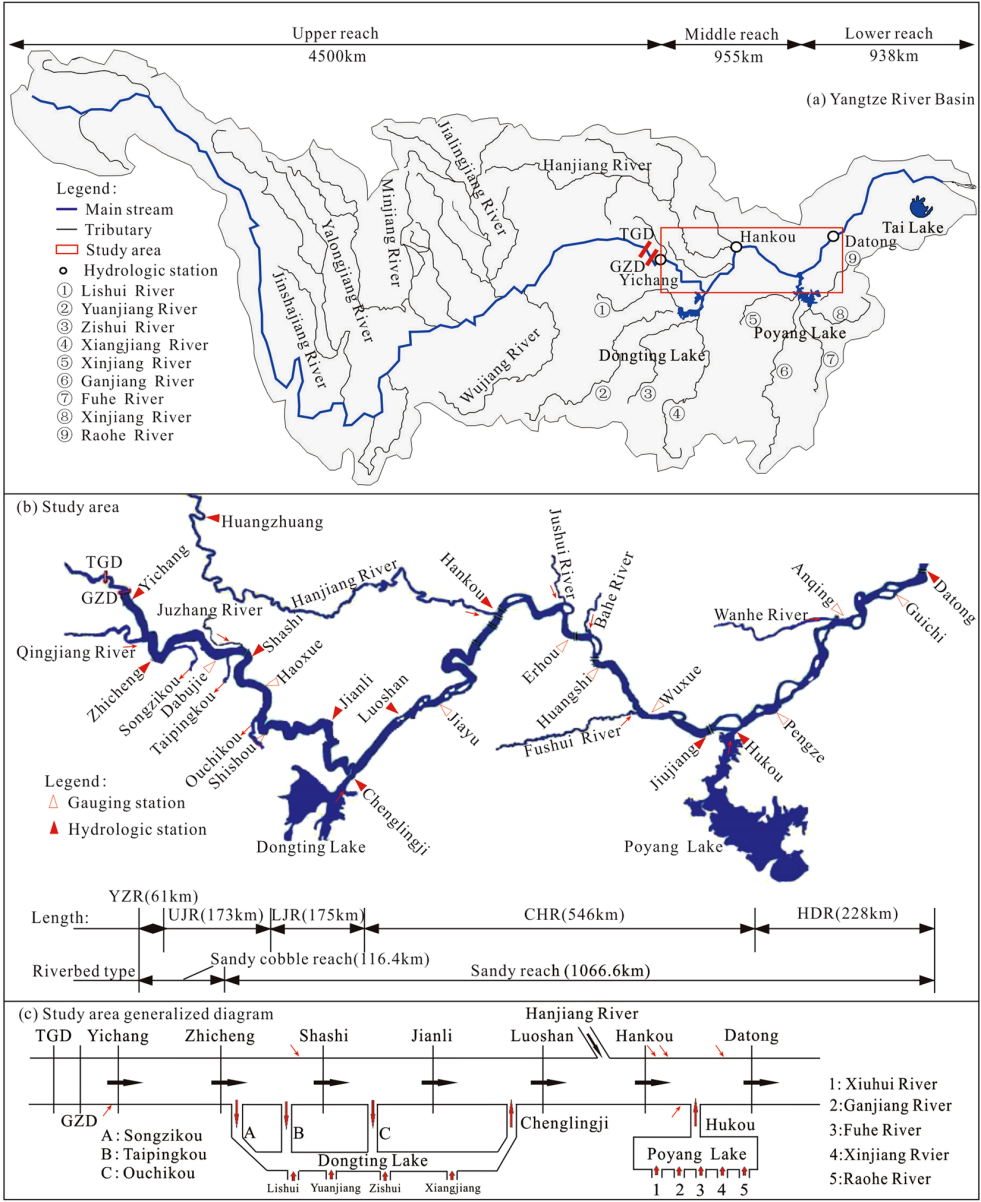
Location map (**a**), and detailed study area (**b**), with hydrologic distribution. Note: The figure shows: (**a**) the geographical location of the Yangtze River reaches, and the study area; (**b**) the study area in more detail, including the length of the reaches, the location of the hydrological stations mentioned in the text, and the compositional information of the riverbed geology; and (**c**) the main water system of the study area. (YZR denotes Yichang to Zhicheng Reach (61 km); UJR denotes Upper Jingjiang Reach (175 km); LJR denotes Lower Jingjiang Reach (173 km); CHR denotes Chenglingji to Hukou Reach (546 km); HHR denotes Hukou to Datong Reach (228 km). Figure 1 Location map (**a**), and detailed study area (**b**), generated using AutoCAD 2009. Then Fig. 1 (**a**), (**b**) and (**c**) were joined by the software of CorelDRAW X6.

On the Yangtze River, the main stream above Yichang is the upper reach, with a length of 4,504 km. The middle reaches extend for 955 km from Yichang to Hukou, in which the section Zhicheng – Chenglingji is known as the Jingjiang Reach, which is further divided into the Upper Jingjiang Reach (UJR) and the Lower Jingjiang Reach (LJR) at Ouchikou. The river below Hukou is the lower reach, which is 938 km long (Fig. 1). The length of the Yichang–Datong reach of the Yangtze River downstream from the TGR is 1,183 km. The Yichang–Dabujie segment is a sandy cobble reach, 116.4 km long, while downstream from Dabujie is a sandy reach of 1,066.4 km (Fig. 1b). The main stream of the river in the study area includes the hydrological stations at Yichang, Zhicheng, Shashi, Jianli, Luoshan, Hankou, Jiujiang, and Datong. Flood diversion channels to Dongting Lake include Songzikou, Taipingkou, and Ouchikou, which are known as the three mouths of Dongting Lake. The Xiangjiang, Zishui, Yuanjiang, and Lishui Rivers converge at Dongting Lake and are known as the Four Rivers of Dongting Lake. The Chenglingji Hydrological Station controls the discharge from Dongting Lake into the main stream of the Yangtze River. The Hanjiang River confluence is controlled by Huangzhuang Station. Hukou Station controls the discharge to the river from Poyang Lake, which is fed by the Xiushui, Ganjiang, Fuhe, Xinjiang, and Raohe Rivers, known as the Five Rivers. The impact of the TGR is most significant in the Yichang–Datong reach, which corresponds to the middle and lower reaches of the Yangtze River.

### Data sources

The hydrological stations involved in this study are Yichang, Zhicheng, Shashi, Jianli, Luoshan, Hankou, Jiujiang, and Datong. Also involved are the three diversion channels to Dongting Lake, and the discharge channels from the Dongting and Poyang lakes, respectively controlled by the Chenglingji and Hukou stations. Runoff rates, flow rates, sediment transport data, and water-level data for the period 1955–2016 were collected from each of the hydrological stations. Deposition, erosion, and cross-section data were collected from the Yichang–Hukou segment from 1987 to 2014. Water-level data were collected along the Yichang–Hankou segment from 1981 to 2016. Information on the depth and width of the shipping channel from 2002 and 2015 was also collected. Data collection times vary, but all data sets end within the past three years, and so include recent information. Table 1 shows the source of each type of data.

**Table 1:** Data sources for hydrological conditions and sediments downstream of the TGD.

Serial number	Hydrologic station	Longitude	Latitude	Data types	Length of time	Source institutions
1	Yichang	111°16′55″	30°41′30″	Water; Sediment; Flow; Water level	1955–2016	Bureau of Hydrology, Changjiang Water Resources Commission; Yangtze River Waterway Bureau.
2	Zhicheng	111°30′00″	30°30′00″			
3	Shashi	112°13′52″	30°18′47″			
4	Jianli	112°52′59″	29°47′00″			
5	Luoshan	113°21′32″	30°40′30″			
6	Hankou	114°17′31″	30°33′56″			
7	Jiujiang	116°01′19″	29°44′38″			
8	Datong	117°36′43″	30°46′41″			
9	Chenglingji	113°07′25″	29°24′52″			
10	Hukou	116°12′55″	29°44′41″			
11	Yichang-Hukou Reach			Amount of erosion and deposition	1981–2015	
12	Shuifu-Yangtze Estuary			Channel depth and width	2002, 2015	

### Flow regulation of the TGR

**Figure 2:**
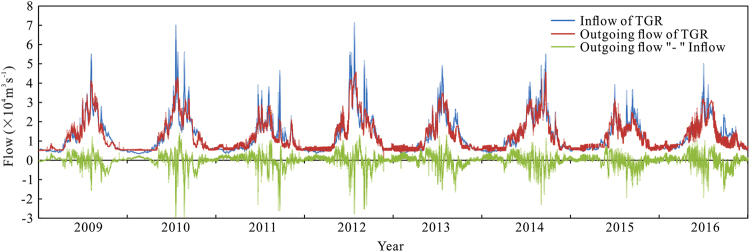
Inflow and outflow during the pilot impoundment stage of the TGR, showing the runoff regulation process (2009–2016).

The TGR began impounding water in June 2003, reaching an impoundment level of 175 m in three separate stages (Fig. 2): (1) June 2003–September 2006; (2) October 2006–September 2008; and (3) October 2008–present. These are distinguished as the cofferdam stage, initial stage, and pilot impoundment stage, with corresponding water retention levels of 135–139 m, 144–156 m, and 145–175 m, respectively. Regulation of water level in the TGR is based on flood peak reduction and drought flow recharge. Thus, during the flood season, the upstream flood peak is drastically reduced to alleviate the pressure of downstream flood prevention, and during the drought season, the discharge is supplemented with the goal of alleviating downstream drought conditions, while increasing channel depth and improving its ecology. Since reaching the 175 m pilot retention stage in 2009, both flood peak reduction and runoff recharge for drought season have become increasingly important. For example, in 2010 the maximum reduction of peak flow reached 30,000 m^3^/s, which ensured that the reservoir discharge rate did not exceed 40,000 m^3^/s. Since 2009, the number of drought season runoff recharge days has increased every year; it reached 189 days during the period between 2015 and 2016, indicating that the flow recharge regulation lasted for more than half of the year (Table 2).

**Table 2:** Summary of water-resources allocation and utilization data during TGR operations.

Impoundment period	Period	Days of making-up water	Total volume of making-up water (×10^9^ m^3^)	Average rising navigable depth (m)	Year	Maximum reduction peak (m^3^·s^−1^)	Times of flood storage	Total flood storage volume (×10^8^ m^3^)
Power generation period with water retained by cofferdam	2003–2004	11	0.879	0.74	2003		Not flood peak	
	2004–2005	Since inflow is high during low-water season, no making-up water operation was conducted			2004		Not flood peak	
	2005–2006	Since inflow is high during low-water season, no making-up water operation was conducted			2005		Not flood peak	
Initial operation period	2006–2007	80	3.580	0.38	2006		Not flood peak	
	2007–2008	63	2.250	0.33	2007	5100	1	10.43
Trial impoundment and operation period	2008–2009	190	21.600	1.03	2008		Not flood peak	
	2009–2010	181	20.020	1.00	2009	16300	2	56.50
	2010–2011	194	24.331	1.13	2010	30000	7	264.30
	2011–2012	181	26.143	1.31	2011	25500	5	187.60
	2012–2013	178	25.410	1.29	2012	28200	4	228.40
	2013–2014	182	25.280	1.26	2013	14000	5	118.37
	2014–2015	82	6.100	1.26	2014	22900	10	175.12
	2015–2016	170	21.760	1.26	2015	8000	3	75.42
	2016–2017	—	—	—	2016	19000	4	227.00

## Changes in the flood and low-flow water levels in the reaches below the TGD

### Variation of the highest and lowest water level

The TGR reduces the peak flow so that the corresponding flood level decreases, while allowing an increase in the low-flow water level during the dry season^27,28^. The highest and lowest water levels in the reaches below the dam after reservoir construction show the combined effects of reservoir runoff regulation, climate change, channel scouring and silting, human activities and other factors. The occurrence of extremely low runoff and water levels cannot be attributed entirely to the reservoir, climate change is not a negligible factor^29,30^.

#### Minimum water levels

**Figure 3:**
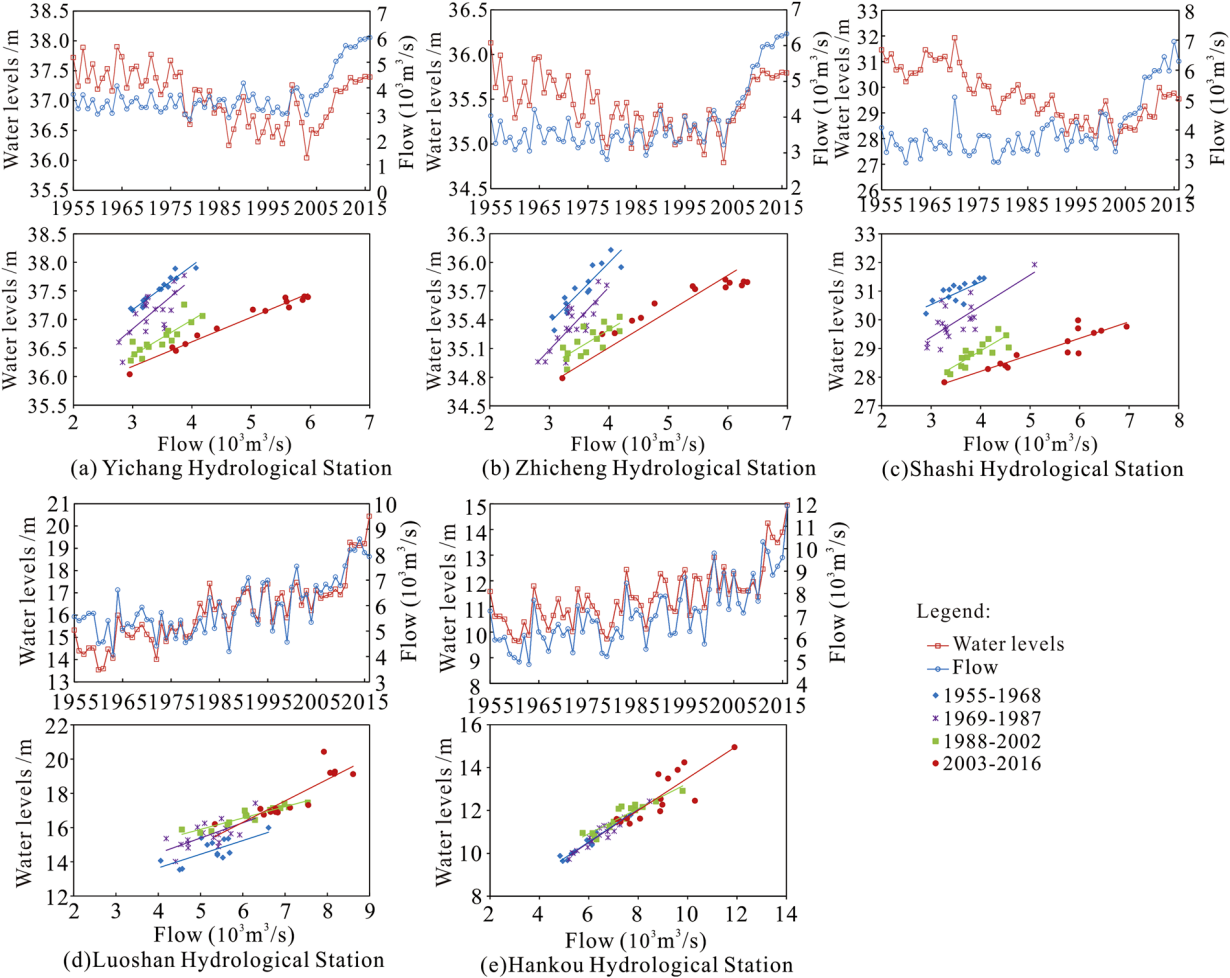
Minimum water levels and corresponding flows at the hydrologic stations downstream of the TGD.

Before construction of the TGR, there was a fluctuating downward trend of minimum water levels at the Yichang, Zhicheng, and Shashi hydrological stations, and the corresponding minimum discharge did not change significantly. After impoundment began at the TGR, the annual minimum water level and minimum flow began to increase (Fig. 3a,b, and c). The Yichang, Zhicheng, and Shashi stations showed a decreasing trend in the corresponding water levels at the same flow rate in the 4 time periods of 1955–1968, 1969–1987, 1988–2002, and 2003–2016. The minimum water levels and minimum flow rates at Luoshan Station and Hankou Station increased steadily, both before and after the TGP was implemented (Fig. 3d and e). This change is related to the decrease of the shunt flow volumes of the three outlets of Dongting Lake along the south bank during the same period^31^. The increase or decrease in the corresponding water levels at the same flow rate at Luoshan and Hankou stations in the 4 time periods of 1955–1968, 1969–1987, 1988–2002, and 2003–2016 was not significant.

#### Maximum water levels

**Figure 4:**
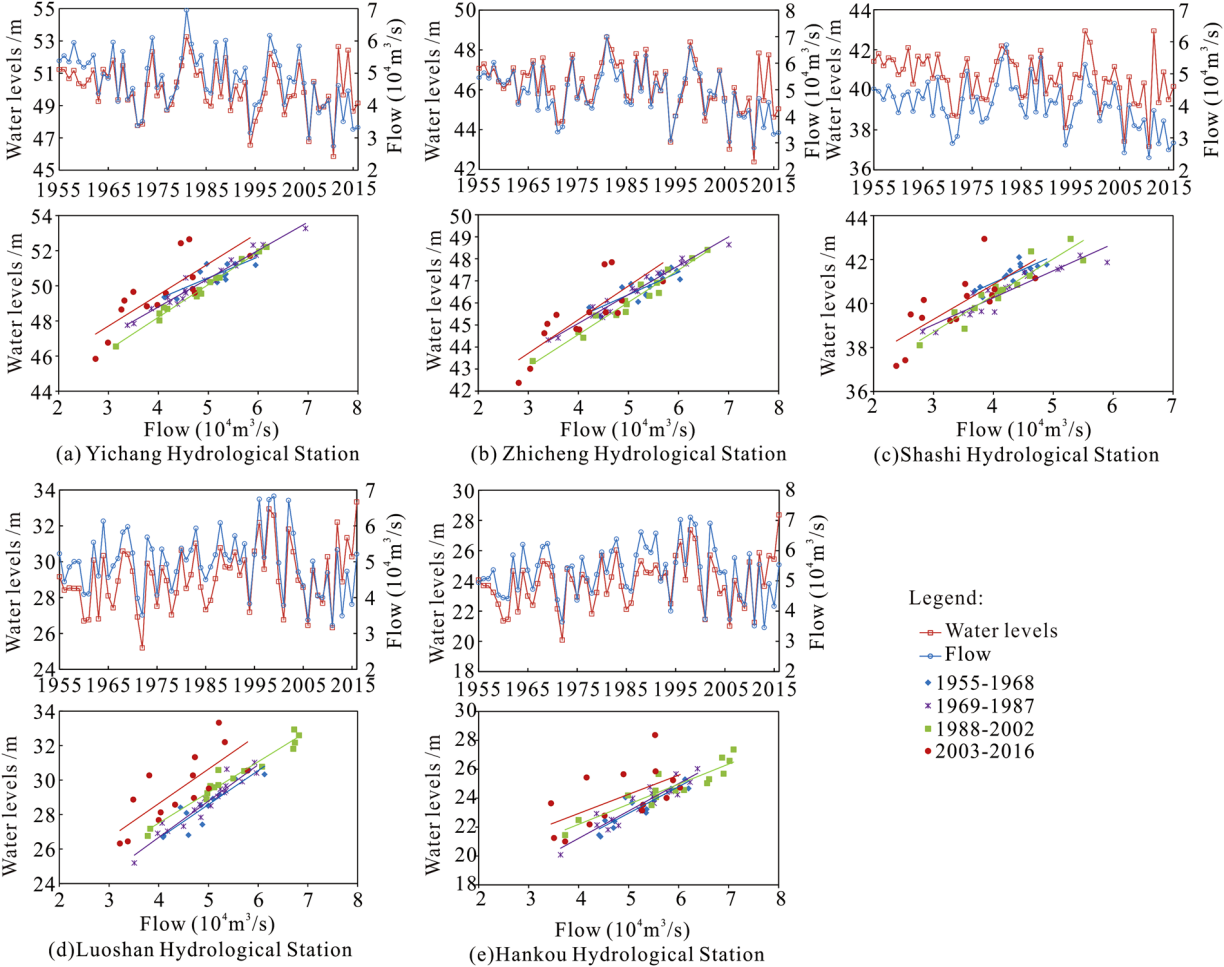
Maximum water levels and corresponding flows at the hydrologic stations downstream of the TGD.

Changes in the maximum water level are shown in Fig. 4. Before the impoundment at the TGR, the highest water level at Yichang, Zhicheng, and Shashi stations showed fluctuating but generally decreasing trends. After impoundment began, there was no significant trend, but the maximum water levels measured were lower than the pre-impoundment maxima. Before construction of the TGR, the maximum water levels at Luoshan Station and Hankou Station increased slightly, but there was no significant elevation change post-construction. A comparison between the water levels during the 2003–2016 period with the water levels corresponding to the same flow rates before construction of the TGR shows that post-construction water levels were all elevated, and the expected decline did not occur.

### Variation of water levels at the same discharge

**Figure 5:**
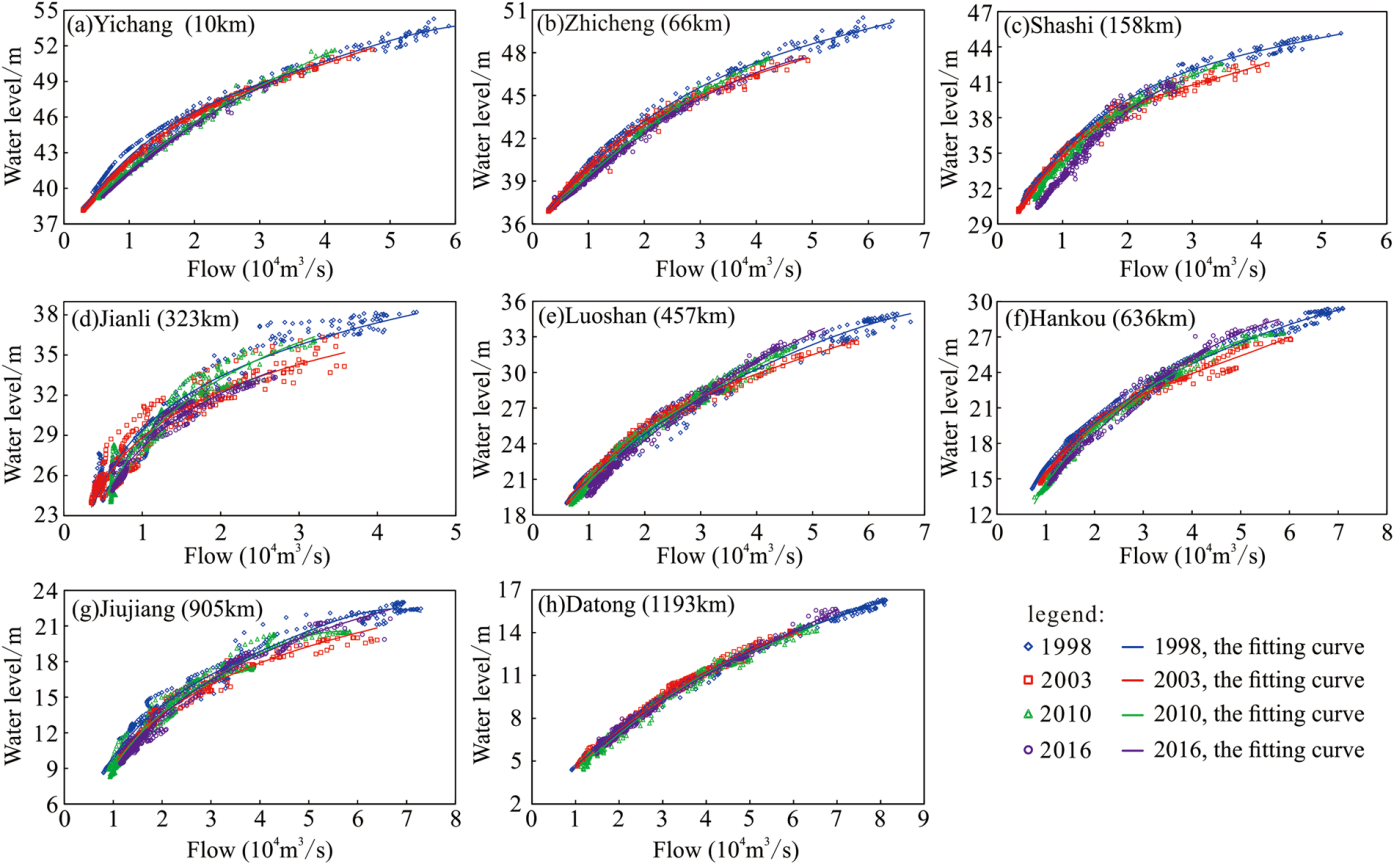
Relationship between the water level and flow in reaches below the TGD.

The years 1998, 2003, 2010 and 2016 were selected as representative years (Fig. 5), during which the water levels of the dry-season discharges dropped, while discharges during the flood season showed an increasing trend. For each station, there is a critical discharge below which a declining trend is observed, while discharges greater than this value increased over time. Comparing the 2010–2016 and 2003–2010 periods, the value of this critical discharge showed a decreasing trend, indicating that the flood characteristics had changed from a high discharge and high flood water level to a moderate flood discharge and high flood water level.

## Reasons for the variation in the flood and low-flow water levels and their influence on the flood control situation and channel water depth

### Analysis of the causes of the variation of the flood and low-flow water levels

Before and after construction of the TGR, the adjustment trends of the low-flow water level and flood level of the river channel were different, suggesting that the influentian factors driving the variation of these water levels were also different. After the impoundment was completed, scouring in reaches below the dam mainly occurred as a result of the basic flow channel, and the expansion of the water area below the low-flow water level was the main factor driving the water-level decline^10^. In addition to exceptional reasons, such as elevation of the erosion datum by sedimentation and flooding at the confluence with tributaries, the increase in river channel resistance due to the coarsening of the bed and increased growth of vegetation in the river channel are the major factors leading to higher flood levels at the same discharge^20,21^. Due to the combined effect of natural and human factors, the decrease in the water level corresponding to the same flow was greater than the increase in water level. The water level with the same flow exhibited a decreasing trend, which should otherwise have increased. Based on this concept, we analysed the effects of changes in any of the influential factors on the drought and flood water-levels under the same flow-rate conditions in the lower reaches of the Three Gorges Dam.

#### Effect of river channel geometry adjustment

**Figure 6:**
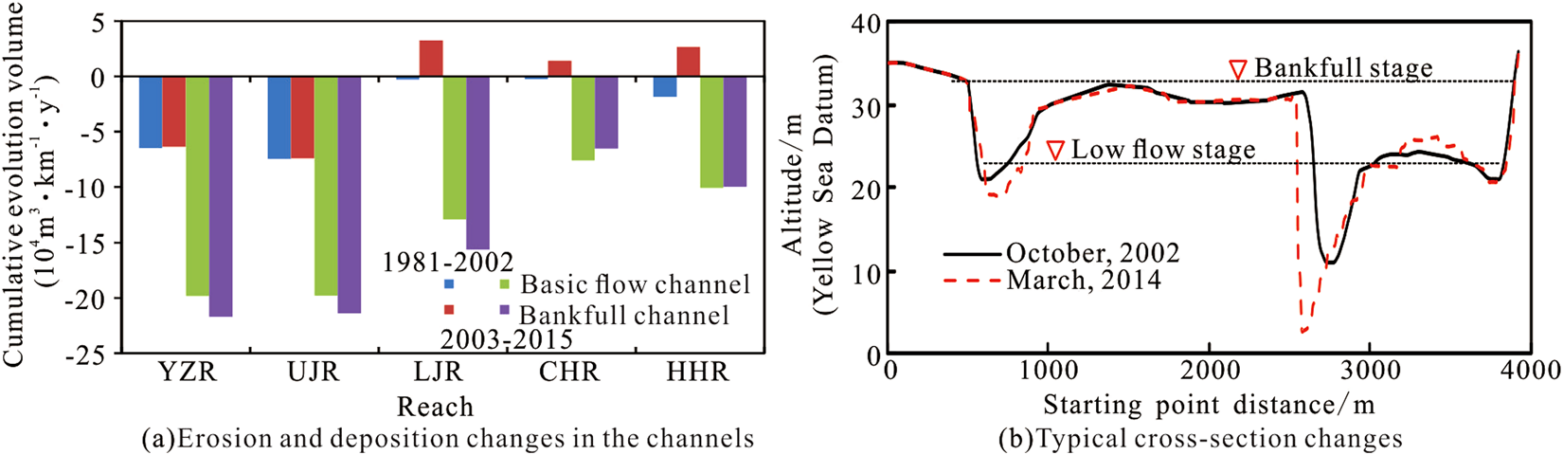
Erosion and deposition changes in the channels of the Yichang–Hukou reach.

According to the statistics of the Bureau of Hydrology, Changjiang Water Resources Commission, from 1981 to 2002, the volume of erosion of the Yichang to Hukou reach from the basic flow channel was 4.91 × 10^8^ m^3^, the sediment volume of the basic flow channel-bankfull channel was 5.05 × 10^8^ m^3^, such that the sediment volume of the bankfull channel was 0.14 × 10^8^ m^3^, and the average annual sediment volume was 67.23 × 10^4^ m^3^ (Fig. 6). During the period from October 2002 to October 2015, the cumulative volumes scoured from the basic flow channel and the bankfull channel in the Yichang–Hukou reach were 15.16 × 10^8^ m^3^ and 15.88 × 10^8^ m^3^, respectively, the annual scouring amounts were 1.16 × 10^8^ m^3^ and 1.22 × 10^8^ m^3^, respectively, and 95.46% of the river erosion occurred in the basic flow channel (Fig. 6). Before construction of the TGR, sediment deposition in the middle and lower reaches of the Yangtze River was the main reason for the increasing flood level at the same discharge^15,32^. After construction of the TGR, erosion in reaches below the dam (Fig. 6) could reduce the flood level in theory, but the trend of river bank erosion was opposite to the elevation of the flood level at the same discharge, indicating that river erosion did not cause the decrease in the flood level. The maximum discharge and water levels at the Yichang and Zhicheng stations, both within the sandy cobble reach, were reduced (Figs 3 and 4). Even if the flood level increased at the same discharge, the pressure on flood control systems in this section was effectively weakened, due to runoff regulation by the TGR. The increase in the area of the river channels in the upper reaches and the reach-scale bankfull channels of the lower reaches of the Jingjiang reach^22^ were conducive to the release of more floods waters. The cross-sectional geometry shows that the shape of the channel above bankfull was not significantly changed (Fig. 6). Dongting Lake and Poyang Lake are downstream of the middle and lower reaches of the Yangtze River, and variations in the lake volume will also affect the main channel flood level. Therefore, when analysing the factors causing the increasing flood level at the same discharge in the main stream of the Yangtze River, we must consider the relationship between the river and lakes, human activities, beach vegetation above the bankfull channel and controls in the watershed.

#### The Influence of River and Lake Diversion

**Figure 7:**
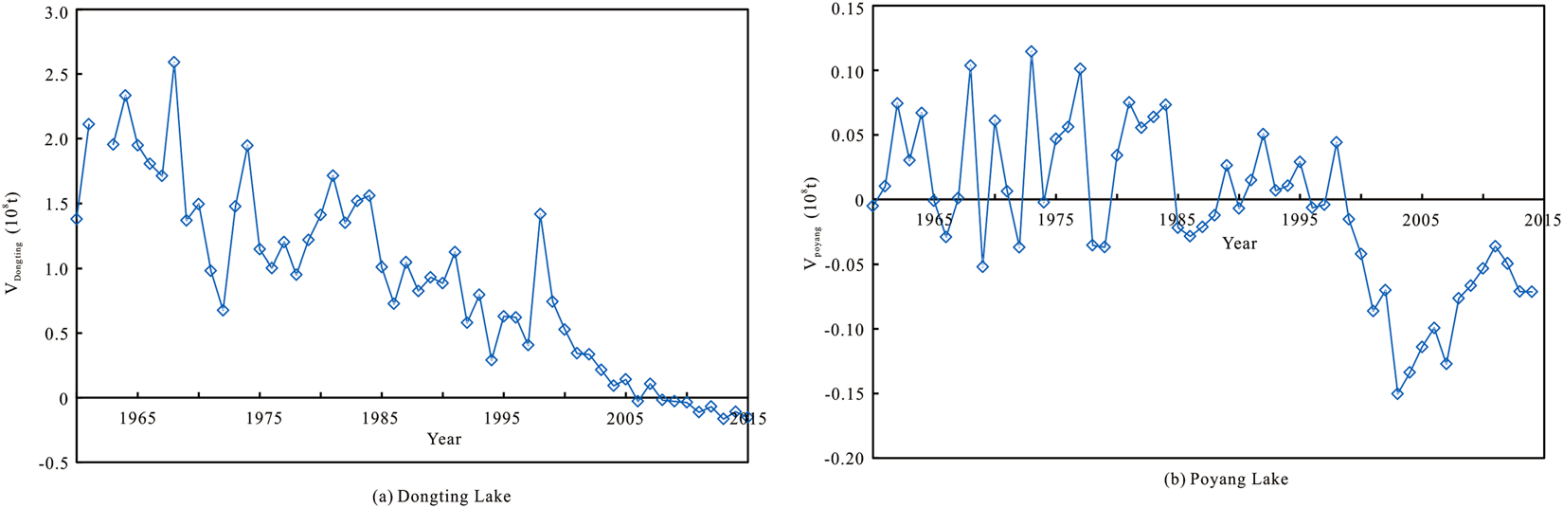
Sedimentation in Dongting Lake and Poyang Lake.

Net siltation occurred in the Dongting Lake region from 1960 to 2006, but the overall balance shifted to scouring from 2007–2015. In the Poyang Lake region, there was alternating siltation and scouring from 1960 to 1999, but from 2000 to 2015, there was a tendency for increased scouring (Fig. 7). Both lakes receive sediment scoured from upstream rivers^15,32^. Before construction of the TGR, the siltation and flooding capacity in the Dongting Lake area decreased, and the flood discharge capacity of the main stream increased, but this did not lead to higher flood levels at the same discharge. Sedimentation of the Luoshan–Hankou reach was the main cause of flood level elevation at the same discharge^15,32^. After construction of the TGR, the trend towards increasing erosion in the Dongting Lake and Poyang Lake was reduced, the area flooded during the flood season was reduced^33^, and the storage volume of the lakes was somewhat increased, reducing the contribution of the lakes to floods and reducing the pressure on flood-control measures along the main stream. Flow rates at the entrances to the river control the influence of the lakes on flooding in the main stream and determine whether flooding in the middle and lower reaches of the river is comprised of upstream flood water or a combination of lake water and upstream flood water. However, the flow rate is not the main determinant of fixed flow-rate flood water levels.

Sedimentation in Dongting Lake and Poyang Lake can be expressed as follows:

1 $${{\rm{V}}}_{{\rm{Dongting}}{\rm{Lake}}}=\mathop{\underbrace{\mathop{\underbrace{{{\rm{S}}}_{{\rm{Songzikou}}}+{{\rm{S}}}_{{\rm{Ouchikou}}}+{{\rm{S}}}_{{\rm{Taipingkou}}}}}\limits_{{\rm{Three}}\,{\rm{mouths}}}+\mathop{\underbrace{{{\rm{S}}}_{{\rm{Xiangjiang}}{\rm{River}}}+{{\rm{S}}}_{{\rm{Zishui}}{\rm{River}}}+{{\rm{S}}}_{{\rm{Ruanjiang}}{\rm{River}}}+{{\rm{S}}}_{{\rm{Lishui}}{\rm{River}}}}}\limits_{{\rm{Four}}\,{\rm{Rivers}}}}}\limits_{{\rm{Input}}}-\mathop{\underbrace{{{\rm{S}}}_{{\rm{Chenglingji}}}}}\limits_{{\rm{Output}}}$$

2 $${{\rm{V}}}_{{\rm{Poyang}}{\rm{Lake}}}=\mathop{\underbrace{\mathop{\underbrace{{S}_{{\rm{Xiushui}}{\rm{River}}}+{{\rm{S}}}_{{\rm{Ganjiang}}{\rm{River}}}+{{\rm{S}}}_{{\rm{Fuhe}}{\rm{River}}}+{{\rm{S}}}_{{\rm{Xinjiang}}{\rm{River}}}+{{\rm{S}}}_{{\rm{Raohe}}{\rm{River}}}}}\limits_{{\rm{Five}}\,{\rm{Rivers}}}}}\limits_{{\rm{Input}}}-\mathop{\underbrace{{{\rm{S}}}_{{\rm{Hukou}}}}}\limits_{{\rm{Output}}}$$

#### Effect of beach vegetation

**Figure 8:**
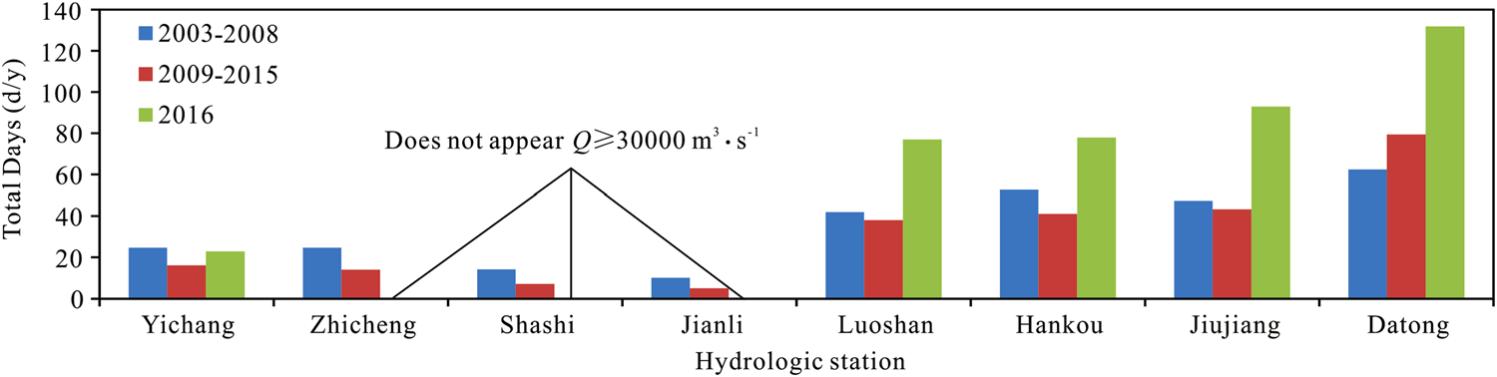
The regulatory effect of the TGR and the change in the number of days of flooding.

Beach vegetation can help prevent erosion of riverbanks and maintain river bed stability^34^. It also increases flow resistance and thereby decreases the speed of the flow, which increases the water level and affects flood prevention to a certain extent^35^. The Mississippi River in the United States flooded in 2011. Flow rates during that flood were lower than those in the floods of 1928 and 1973^3^. Lush vegetation at higher elevations in the flood plain (corresponding to high beach areas) caused backwater that further increased the flood water level^4^. This led to localized “medium flood flow rate, high flood water level” conditions. Figure 8 shows the number of days per year that the flow rate exceeded 30,000 m^3^/s in 2009–2015 and in 2016 after water was stored in the TGR compared to that in 2003–2008. Over the 2009–2015 period, the number decreased slightly overall, but in 2016, it increased considerably at Luoshan and further downstream (Fig. 8). Flooding occurred in the lower reaches of Luoshan and in the middle reaches of the Yangtze River in 2016, and water inundated the floodplain for a longer period of time. Prior to this, the number of days of flooding was low, and the vegetation on the beach was relatively lush, which increased the river flood capacity to flood level. In the Zhicheng – Chenglingji River section, due to the combined effects of peak cutting by the TGR and diversions to Dongting Lake, the floodplain rarely flooded, and thus Jingjiang reach flood control security was guaranteed. In 2016, the flow rate in the Hankou – Jiujiang reach was high, due to flows from the Daoshui, Jushui, Bahe, and Xishui tributaries. These tributaries caused the flow rate in the main stream to rise by as much as 24,800 m^3^/s (measured on June 30, 2016, as the difference between the flow rates at Jiujiang and Hankou stations). This partially explains why the flood water level at Hankou was high in 2016. The channel-boundary node protruding from river bank of the Wuhan–Jiujiang reach is a hindrance to flooding^36^, and when the Wuhan–Jiujiang reach discharge is ≥50,000 m^3^/s, the Tianjiazhen node blocks the discharge of upstream flooding for 2 to 3 days^37^, which also leads to a high flood level capacity, and is one of the reasons for the high flood level of this section.

#### Influence of shoreline control, waterway management, and other human activities

The Jingjiang reach is a major flood control region in the middle reaches of the Yangtze River and is regarded by many to be the most dangerous part of the Yangtze, as river bank collapses occur occasionally^22^. There are ports, wharves, bridges, and scenic works all along the banks of the middle and lower reaches of the Yangtze River, and these occupy portions of the flood control zone and narrow the flood channel. After the floods of 1998, the water conservancy department strengthened and improved the prevention and control embankments on the middle and lower reaches of the Yangtze River, and the flood control capacity of the embankment was increased by 0.51–3.00 m (Table 3). The total number of embankment collapses in 2016 was only 0.51% of that in 1998^18^, and there was no greater risk (Table 4), indicating that embankment reinforcement and improvement can effectively reduce flooding.

**Table 3:** Embankment flood control standards, compared with those before 1998.

Flood control ability	Shashi	Jianli	Chenglingji	Hankou	Jiujiang	Anqing
Before 1998 (m)	44.49	35.70	32.30	27.12	20.00	16.50
Current situation (m)	45.00	36.57	34.40	29.73	23.00	18.74
Height elevation (m)	0.51	0.87	2.10	2.61	3.00	2.24

**Table 4:** Disasters on the embankments of the Yangtze River in 2016 compared with those in 1998^18^.

Year	Project	Number	Hubei	Hunan	Jiangxi	Anhui	Jiangsu
1998	Number of disasters	9405	3133	1324	4162	640	146
	Number of severe disasters	698	402	97	41	115	43
2016	Number of disasters	50	26	5	12	0	7
	Number of severe disasters	0	0	0	0	0	0
Ratio of the number of disasters between the two years (%)		0.53	0.83	0.38	0.29	0.00	4.79

The middle and lower reaches of the Yangtze River have always had the reputation of being a “golden waterway”. During the period from 2003 to 2015, the Yangtze River Waterway Bureau implemented a targeted waterway regulation project. For the sand and cobble river sections, the project goal was to protect the bottom of the river, to prevent the water level in the channel from dropping as a result of further erosion. For sandy river sections, the goal was mainly to protect the bank beaches and central beaches, and protection or adjustment works were carried out at the low beaches of the centre and sides of the Yangtze River and its tributaries. At the same time, to improve the boundary stability, shoreline reinforcement or revetment works were implemented, and the flood storage capacity was somewhat improved by the comprehensive engineering project. In a June 2015 report, the Changjiang River Scientific Research Institute (CRSRI)^38^ showed that after remediation of 29 shipping obstructions, 10 sections of beach had a maximum hydraulic resistance of 5–12.2%, and 19 sections of beach had a hydraulic resistance of 0–5%. In seven sections of beach, maximum flood water levels were elevated by 5–12.4 cm, and in 22 sections of beach, maximum flood water levels were elevated by 1–5 cm. Single projects have limited impact on overall flood control, but the combined influence of all bridges and wharves has a significant impact on flood water levels and adversely affects flood control in the channel^39,40^.

#### Effect of river bed coarsening

A rougher river bed increases a river’s hydraulic resistance, and raises water levels. After water was stored in the TGR, the river bed downstream became rougher^23,24^. The median grain size (D_50_) of the surface of the river bed between Yichang and Zhicheng increased by a factor of 48, i.e., from 0.638 mm in December 2003 to 30.4 mm in October 2010. Between Zhicheng and Dabujie, the D_50_ value increased by a factor of 20. The roughness increased by 91%, 65%, 3%, and 2% in reaches below the TGD from Yichang to Zhicheng (distance to Yichang is approximately 61 km), from Zhicheng to Dabujie (61 km to 116.4 km), in the upper Jingjiang reach (116.4 km to 319 km), and from Chenglingji to Hukou (319 km), respectively (Table 5).

**Table 5:** Roughness changes caused by riverbed coarsening^46^.

Riverbed composition	Gravel and pebble river		Sandy River	
Reach	Yichang-Zhicheng	Zhicheng-Dabujie	UJR	Chenglingji-Hukou
Increasing amplitude (%)	91	65	3	2

Mathematical model calculations^40^ show the following: in the pebble and gravel Yichang–Dabujie reach of the river, after coarsening of the river bed, the water levels corresponding to Yichang flow rates of 5,000 m^3^/s, 10,000 m^3^/s, 23,000 m^3^/s, and 35,000 m^3^/s increased by an average of 1.57 m, 2.04 m, 2.7 m, and 3.3 m, respectively. These values are higher than the actual drop in the water level due to the increased channel depth corresponding to each flow rate increase. This explains how coarsening of the river bed effectively mitigated low water-level drops in this pebble and gravel section of the river. In the sandy reaches downstream from Dabujie, the average increases in the water level corresponding to Yichang flow rates of 5,000 m^3^/s, 10,000 m^3^/s, 23,000 m^3^/s, and 35,000 m^3^/s were 0.13 m, 0.11 m, 0.16 m, and 0.16 m, respectively. These increases were limited relative to the effects of undercutting.

Based on the above analysis, changes in the drought water-level for the same flow were as follows: before and after the impoundment of the TGR in the Yichang-Zhicheng and Upper Jinjiang Reach, river channel erosion was the main cause of the decreased drought water-level; in the Lower Jingjiang Reach, Chenglingji-Wuhan, and Wuhan-Hukou reach, river channel sedimentation before the impoundment developed into erosion after the impoundment, and because the decrease in water level caused by channel erosion was similar to that caused by channel coarsening, there was no obvious trend in the same-flow drought water level. The changes in the flood water-level under the same flow-rate were as follows: Before and after the abovementioned impoundment, the Yichang-Zhicheng and Upper Jingjiang Reach exhibited a continuous erosion trend; the Lower Jingjiang, Chenglingji-Wuhan, and Wuhan-Hukou Reach changed from sedimentation to erosion; and the trend in the flood water level under the same flow-rate was inconsistent with the channel erosion trend, which indicated that channel erosion was not the main reason for the increased flood water-level under the same flow-rate.

The confluence points of the Dongting and Poyang Lakes only influenced water levels close to the intersections and had a minor influence on the mainstream flood water-levels under the same flow-rate. Moreover, lake erosion improved the regulation of lakes and weakened the contribution of Lake Flood water to the mainstream runoff, thus helping to alleviate the flood control pressure of the Yangtze River mainstream. After the impoundment of the TGR, the duration of the flow rate exceeding the bankfull elevation downstream of the dam decreased, increasing the exposure time of the area above the bankfull channel and, in turn, allowing the shore vegetation to flourish. This resulted in an increased flood water-level under the same flow-rate due to the backwater effect.

However, this is not unique to the Yangtze River. The importance of this effect is illustrated by the Mississippi River floods in 2011. The combined effect of river channel erosion, waterway engineering, and river regime control engineering caused riverbed coarsening, which further accelerated the increase of the same-flow flood water-level. Substantial waterways, river regime control, and shoreline engineering were implemented in the middle and lower reaches of the Yangtze River, which significantly reducing the floodwater river width. Although the effects of individual projects are small, the combined effect of many can contribute towards the upward trend in the same-flow flood water-level.

### Relationship between changes in low water levels and siltation and scouring of the channel

**Figure 9:**
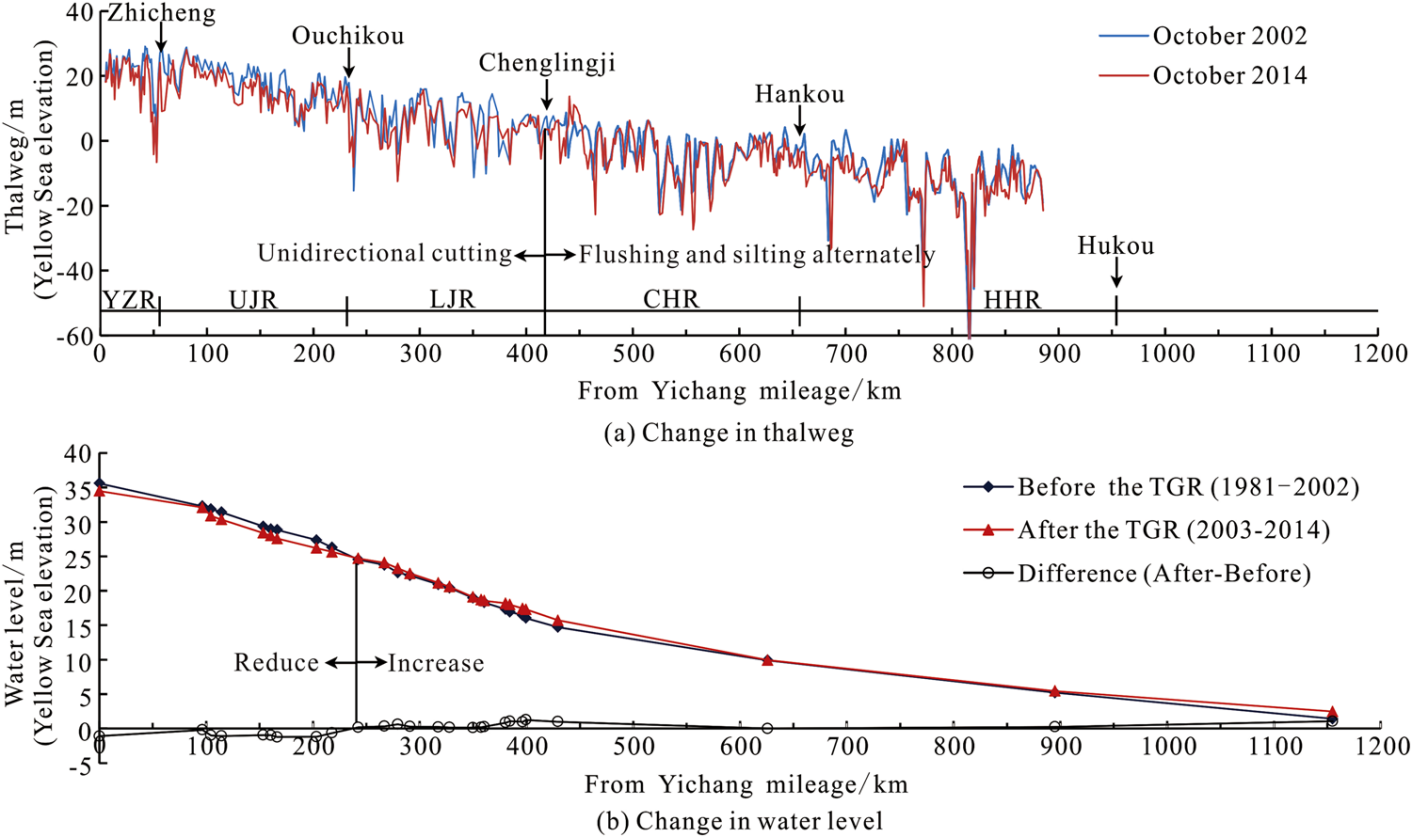
Downstream thalweg and water level elevations before and after construction of the TGR. Note: (**a**) depths are from a selected number of cross-sections along the Yichang – Hukou river section, with a spacing of generally 200–500 m. The thalweg depth is the deepest point of the cross-section. In the case of multiple waterways, the main channel of the multiple waterways is selected; (**b**) water levels (98% highest water level) measured relative to the waterway datum during 1981–2002 and 2003–2014, and the difference in elevation between these periods.

The predict show that after 40 years (2005–2045) of impoundment^13^, when the flow (Q) at Yichang Station 20,000 m^3^/s, the low-flow water level in the 700 km reach below the dam will have a decreasing trend. An examination of the depth of the channel in a 410 km section of the river downstream from the TGR in October 2014 showed an average undercutting of 1.50 m compared with October 2002. Further downstream, alternating siltation and scouring occurred at several locations during this same period (Fig. 9a). On average, the low-flow water level in the 240 km downstream from the TGR was 1.10 m lower in the 2003–2014 period compared with 1981–2002; further downstream, the low-flow water levels rose on average when compared with the low-flow water level from 1981–2002. Undercutting was concentrated in the Yichang–Zhicheng reach and in the upper and lower sections of the Jingjiang reach, and low water level decreases mainly occurred in the Yichang–Zhicheng reach and the upper section of the Jingjiang reach (Fig. 9b). In the immediate future, undercutting in the lower sections of the Jingjiang reach should be controlled to prevent low water levels from dropping.

**Figure 10:**
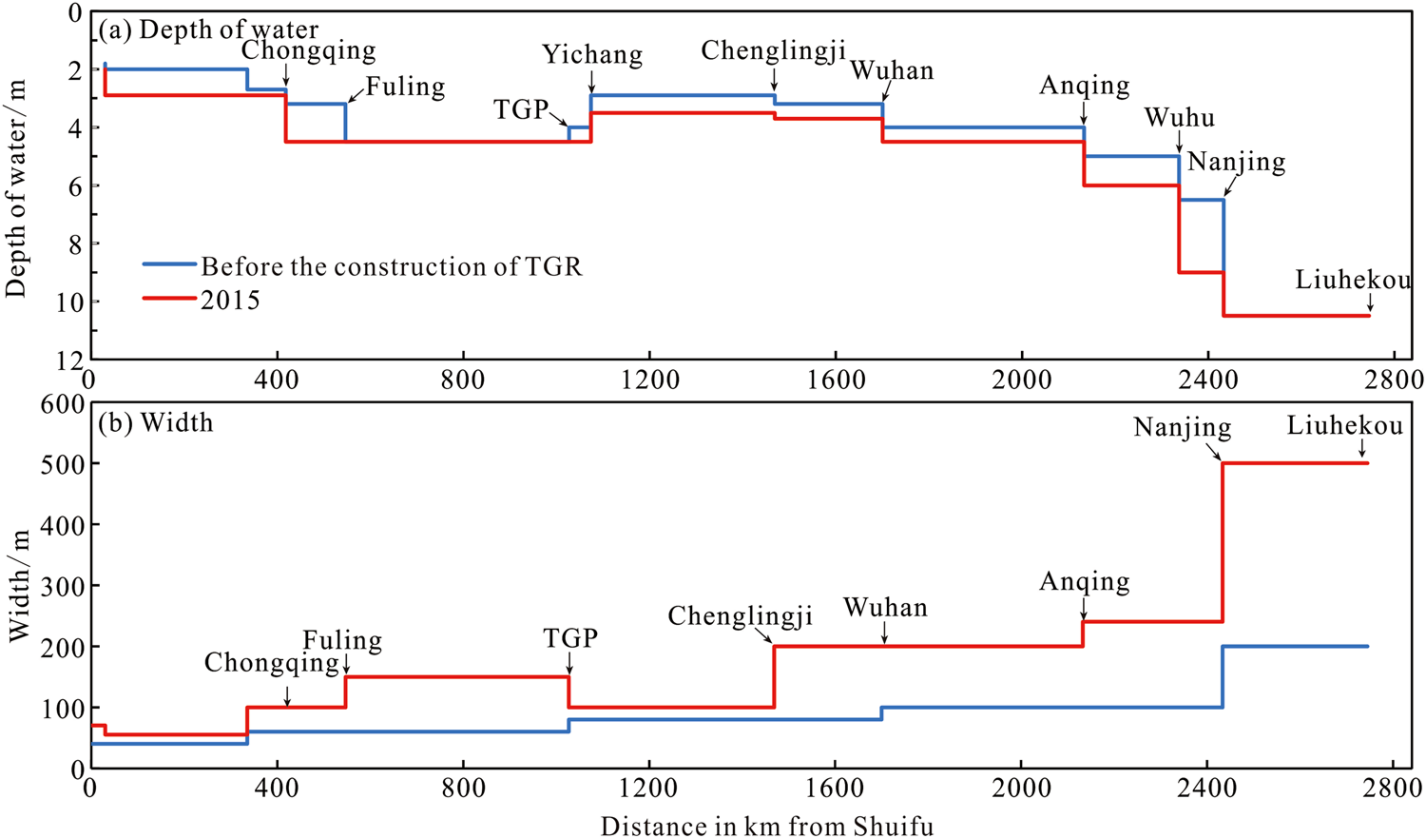
Change in the depth and width of waterway from Shuifu to Yangtze Estuary. (Note: The water depth and width of the waterway are the values of the main waterway).

Since water has been stored at the TGR, the depth of the waterway has been controlled mainly by erosion. To prevent adverse effects, such as shoreline collapse, beach and shoal atrophy, and reduction of flow in the main waterway during the dry season, the Yangtze Waterway Bureau carried out systematic waterway remediation work in the middle and lower reaches of the Yangtze from 2003 to 2015. As a result, the depth of the waterway increased by 0.50 to 0.60 m in the Yichang–Chenglingji, Chenglingji–Wuhan, Wuhan–Anqing, Anqing–Wuhu, and Wuhu–Nanjing reaches, from 2.9 m, 3.2 m, 4.0 m, 4.5 m, and 6.0 m (2003) to 3.5 m, 3.7 m, 4.5 m, 6.0 m, and 9.0 m (2015), respectively (Fig. 10). The width of the waterway also increased significantly. Thus, by 2015, the dimensions of the waterway had already increased to the target dimensions for 2020^41,42^.

### Impact of changing flood water levels on flood control conditions in the middle reaches of the Yangtze River

**Figure 11:**
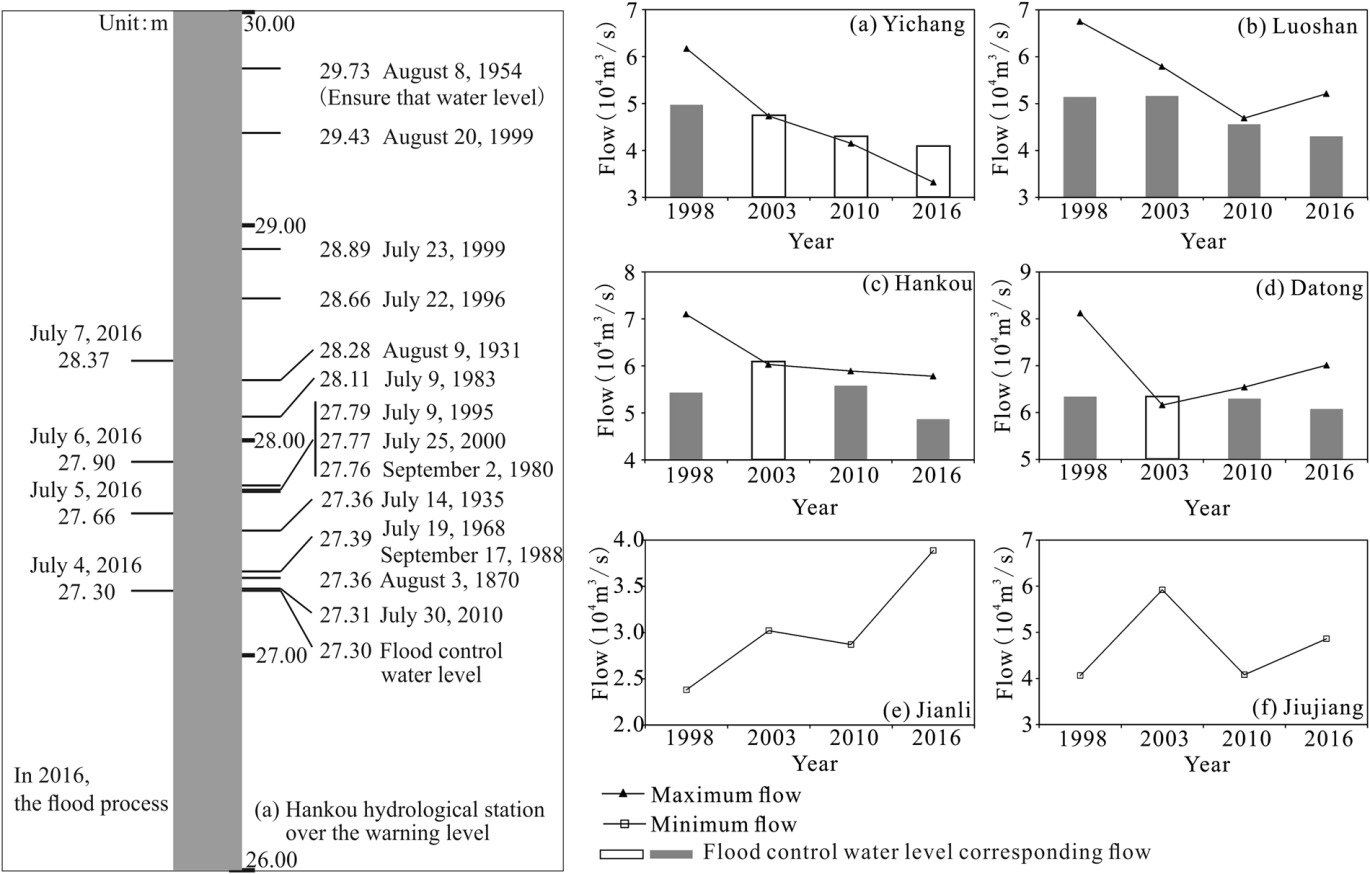
Flood control losses in the middle and lower reaches of the Yangtze River. Note:  Indicates that situations exceeding the flood-alarm level did not occur in this year, so the discharge corresponding to the warning water level must be obtained from fitting the value of the Q - h curve.  Indicates that situations exceeding the flood control water level did occur in this year, and the discharge corresponding to the warning water level is the measured value.

Wuhan is the key city for flood control in the middle reaches of the Yangtze River. Using Hankou Station as an example, during the period from 2003 to 2016, the flood control water level was exceeded in both 2010 and 2016. The highest water level in 2010 exceeded the flood control water level by 1 cm, while the water level exceeded the flood control water level by 107 cm on 7 July 2016; this is the 5^th^ highest water level since 1870. The discharge corresponding to the flood control water level in different years has recently been recalculated (Fig. 11), and the discharge at the flood control water level at Yichang Station, Luoshan Station, Hankou Station and Datong Station declined, whereby the flood-alarm discharge in 2016 compared with 1998 reduced by 8,800 m^3^/s, 8,400 m^3^/s, 5,600 m^3^/s and 2,600 m^3^/s, respectively. The discharge in 2016 also showed a decreasing trend compared with 2003. Jianli and Jiujiang stations were affected by the backwater effect at the outflow points of both Dongting and Poyang Lakes. The water-level and flow-relationship curve (Fig. 5) shows a wide flow-fluctuation range corresponding to flood control warning levels, and the statistical analysis shows that the minimum flow rate reached the flood control warning level (Fig. 11). On the other hand, the flow rate at Jianli station exceeded the minimum flood control warning level, showing an increasing trend, which indicates that the influence of Dongting Lake inflow on the mainstream water level weakened. This can be attributed to the combined effect of the Three Gorges Reservoir regulation and the increase in the lake basin capacity. The minimum flow at Jiujiang station also exceeded the flood control warning level due to the increasing trend. However, compared with Jianli station, the trend was relatively weak. Due to the large distance between the station and the TGR, the effect of reservoir regulation was relatively weak, but the increased lake basin capacity was beneficial in reducing the mainstream runoff. In the near-dam section at Yichang Station, due to the peak-cutting effect of the TGR, the discharge flow was drastically reduced, and the number of days exceeding the flood-control water level was also reduced. Before construction of the TGR, siltation of the Luoshan reach was the main reason for the increasing flood level^43,44^. However, due to river erosion in the Luoshan-Hankou reach after construction, there was an increase in vegetation and bed resistance and an obvious increase in water capacity, which was the main reason for the change in flood elevation at Luoshan Station, before and after construction of the TGR. A comparison of the periods 2003–2013 and 2000–2002 indicated that when the flow rate at Hankou Station was 50,000 m^3^/s, the corresponding increase in the water level was 0–1.0 m^17^. A comparison between 2016 and 2003 showedthat this elevation trend did not decrease, which is not conducive to flood control in Wuhan.

In July 2016, a major regional flood occurred in the middle and lower reaches of the Yangtze River. Through the use of the TGR and upper reaches of the cascade reservoirs, 22.7 billion m^3^ of flood water was intercepted and stored. Consequently, the water levels in the Jingjiang reach, the Chenglingji area, and the lower reaches of Wuhan were reduced by 0.8–1.7 m, 0.7–1.3 m, and 0.2–0.4 m, respectively, and the length of the section was reduced decreased by 250 km, which effectively reduced the pressure on flood-control measures in the Chenglingji reach and Dongting Lake area in the middle reaches of the Yangtze River. Scenarios exceeding the alarm level in the Jingjiang reach and flood diversion to the Chenglingji area were avoided, the safety of the population of the Jingjiang reach was secured, and the integrity of the Yangtze River embankment was guaranteed.

Analysis of water level data from 38 rivers in the United States indicates that dam construction resulted in a reduction in the average annual flood peak discharge of 7.40% to 95.14%^45^. According to the TGR Dispatching Rules, the TGR will regulate small and medium floods^16^, which will effectively alleviate the flood risk in the Yichang–Luoshan River reach. After the flood control standard at the Jingjiang embankment is improved, pressure on flood-control measures on this river section will be greatly reduced. The combined regulating process of the TGD and its upstream cascade reservoirs can not only stop floods with a large flow rate, but also gradually stop floods with low and medium flow rates, resulting in a decrease in the peak value of the outflow during the flood season in the TGD. After a continuous long period, the large flood processes downstream of the TGD will be lost, and the flow process will tend to become uniform throughout the year. With no shaping of river channels due to periods of large flow rates, the flood capacity of the river channels will decrease^16^. Once severe floods occur, especially regional floods in the middle and lower reaches of the TGD due to integrated effects of Dongting Lake, Poyang Lake, Hanjiang River and other tributaries and local rainfall, these floods will be less regulated by the TGD. Without long-term shaping of flood river channels by large floods, the vegetation in the high beach areas will become lush, and silt will gradually accumulate, resulting in increases in integrated resistance of river channels, and the effective flood control capacity will be further reduced^16^.

After the impoundment of the TGR, floods similar to those in 1954 and 1998 did not occur in the middle and lower reaches of the Yangtze River. However, as the discharge corresponding to the flood-control water level reduces, a high flood discharge – high flood level gradually transforms into one of moderate flood discharge – high water level, which should raise concerns of increased flooding. For the near future, issues of flood control and disaster reduction are still highly significant along the Yangtze River, and flood control is still the primary task of development and protection in this area. We should continue strengthening the comprehensive Yangtze River flood control and disaster reduction system and its management, as well as speeding-up the transformation from flood control to flood management and other measures to address flood security issues.

## Conclusions

Construction and operation of the TGP have been studied by many researchers. The effect of reservoir operation on the flood and low-flow water levels in the lower reaches and related issues have been the focus of the river management and waterway management departments. Through analysis of measured data from the reaches below the TGD over the period 1955–2016, the following conclusions have been drawn:

1. The low-flow water level at the same discharge in the reaches below the TGD declined, an effect also observed in downstream water levels of other large dams around the world. Due to the compensating effect of the reservoir during dry seasons, the discharge at low-flow water levels is increased and the lowest water level showed an increasing trend. At the channel scale, the decline in the low-flow water level in the reaches below the TGD is less than the undercutting value of the basic flow channel. Governed by the action of such a large-scale waterway regulation project, water depths in the reaches below the dam have been improved, and the proposed objective for 2020 has been achieved five years in advance.
2. The flood water level at the same discharge increased slightly in the channels below the TGD. The flood characteristics had a tendency to change from a high flood discharge-high flood level to a moderate discharge-high flood level, which is not conducive to flood safety. Peak cutting by the reservoir reduced the highest water levels downstream. For example, during the regional flood of July 2016, the flood height in the reaches below the dam (based on flood heights and flows prior to dam construction) was reduced by 1.7–0.2 m; but the relative magnitude of the reduction declined downstream. The scouring of Dongting Lake and Poyang Lake and the main channel erosion allowed for additional reduction of the flood water level at the same discharge. Increased vegetation, coarsening of the riverbed and human activities have led to increased flood levels at the same discharge in the reaches below the dam, although the flood level at the same discharge in this area was expected to decline.

In the future, the TGR and the upstream cascade reservoirs will adopt a joint dispatching method, and the sediment volume downstream of the dam will be kept at a low level. The continuous flooding and erosion of the river will tend to decrease the low-flow water level, and insufficient water depth in the waterway caused by the drop of the low-flow water level should be controlled and avoided. The joint dispatching of the TGR and the upper reach cascade reservoirs will play a more prominent role in reducing flood peaks, which will reduce pressure on flood control measures downstream of the dam. Nonetheless, the current elevation of flood water level at the same discharge should be a focus of concern.
